# Freshwater Algal Toxins: Monitoring and Toxicity Profile

**DOI:** 10.3390/toxins12100653

**Published:** 2020-10-13

**Authors:** Angeles Jos, Ana M. Cameán

**Affiliations:** Area of Toxicology, Faculty of Pharmacy, University of Sevilla, C/Profesor García González 2, 41012 Sevilla, Spain; camean@us.es

Climate change and human activities are more and more affecting the dynamics of phytoplankton communities. Among them, cyanobacterial abundance has increased disproportionately relative to other phytoplankton, and this trend is likely to continue in the coming decades. This fact has deleterious effects on ecosystem biodiversity but also adversely affects drinking water supplies, livestock watering, crop yields, aquaculture, etc. Thus, the proliferation of cyanobacterial blooms represents an economic issue and, more importantly, human and animal health risks due to the common production of potent toxins, cyanotoxins. Moreover, these risks are increased due to their accumulation potential and their transference to the food chain.

In spite of the worldwide increasing occurrence of cyanotoxins, they are still underestimated in regulations, with Microcystin-LR (MC-LR) as the main one (and in many cases the only one) considered. However, risk management of cyanotoxins is only possible after a thorough risk evaluation, and for that purpose, toxicity and exposure data are required. Thus, occurrence and monitoring information is of key importance, and new data in relation to the conditions that favor cyanobacterial growth and cyanotoxins production are welcome in order to prevent their appearance.

On the other hand, in regard to toxicity, the scientific literature shows already a wide array of adverse effects cyanobacterial toxins can induce. However, there are still health consequences not investigated deeply enough as well as many data gaps in different aspects regarding different targets of cyanobacteria toxicity, from plants to animals and humans. Thus, the aim of this special issue was to gather new studies that could contribute in the risk evaluation process of cyanotoxins. This goal was achieved with a compilation of 11 articles (10 research papers and a review).

Among the articles focused on monitoring issues, Hartnell et al. [1] investigated the cyanobacterial abundance and the MC profiles in two southern British lakes. They could not correlate the elevated MC concentrations found with the number of cyanobacterial cells, but the linear regression analysis performed suggested that temperature and dissolved oxygen could explain the variability of MC across both reservoirs. They concluded that there is a need to develop inclusive, multifactor holistic water management strategies to control cyanobacterial risks in freshwater bodies.

Taranu et al. [2] applied multivariate canonical analyses and regression tree analyses to identify how different congeners (MC-LA, -LR, -RR, and -YR) varied with changes in meteorological and nutrient conditions over time and space. They found that MC-LR was associated with strong winds, warm temperatures, and nutrient-rich conditions, whereas MC-LA, for example, tended to dominate under intermediate winds and nutrient-poor conditions. Thus, the knowledge of the environmental factors leading to the formation of different MC congeners in freshwaters is necessary to assess the duration and the degree of toxin exposure under future global change.

Tamele and Vasconcelos [3] performed a review about the MCs incidence in the drinking water of Mozambique. This report showed that the few studies done in Maputo and Gaza provinces indicated the occurrence of MC-LR, -YR, and -RR at concentrations above the maximum limit recommended by the World Health Organization. Authors evidenced the need to implement an operational monitoring program of MCs in order to reduce or avoid the possible cases of intoxications since the drinking water quality control tests in the country do not include a MCs test.

Other aspects were covered by Perez and Chu [4], LeBlanc et al. [5], and Galetović et al. [6]. Perez and Chu [4] focused their study on zinc metal resistance and stress response in a toxigenic cyanobacterium, *Microcystis aeruginosa* UTEX LB 2385, by monitoring cells with ZnCl_2_ treatment. Among their results, they found that *M. aeruginosa* UTEX LB 2385 was able to survive ZnCl_2_ concentrations of up to 0.25 mg/L, with increasing biomass through 15 days. A persistent yield of the cyanotoxin MC-LR (µg/cell) was observed in all ZnCl_2_ treated cells by 15 days, indicating that this cyanotoxin remains present in the environment even with low cell concentrations.

Leblanc et al. [5] isolated a new MC, [D-Leu^1^]MC-LY, and other related ones, from *Microcystis aeruginosa* strain CPCC-464. The compound was characterized by ^1^H and ^13^C NMR spectroscopy, liquid chromatography–high resolution tandem mass spectrometry (LC–HRMS/MS), and UV spectroscopy. Moreover, [D-Leu^1^]MC-LY showed a potency similar to MC-LR in the protein phosphatase 2A inhibition assay. The authors concluded that [D-Leu^1^]-containing MCs may be more common in cyanobacterial blooms than is generally appreciated but are easily overlooked with standard targeted LC–MS/MS screening methods.

Finally, regarding monitoring-related aspects, Galetović et al. [6] reported the absence of cyanotoxins in Llayta, edible Nostocaceae colonies from the Andes Highlands, by using molecular and chemical methods. Thus, they concluded that Llayta could be considered a safe ingredient for human consumption.

Cyanotoxins toxicity has been an important topic in this special issue. The study of Llana-Ruiz-Cabello et al. [7] explored the susceptibility of two green vegetables, spinach and lettuce, to the cyanotoxins MC and cylindrospermopsin (CYN), individually and in mixture. The study revealed growth inhibition of the aerial part in both species when treated with 50 µg/L of MC, CYN, and CYN/MC mixture. MC showed to be more harmful to plant growth than CYN. Additionally, CYN, but not MC, was translocated from the roots to the leaves. CYN and MC affected the levels of minerals, particularly in plant roots.

Foss et al. [8] described a case report in which MC intoxications of canines were diagnosed through interpretation of clinicopathological abnormalities, pathological examination of tissues, microscopy, and analytical MC testing of antemortem/postmortem samples. The described cases represent the first use of urine as an antemortem, non-invasive specimen to diagnose MC toxicosis. Authors concluded that antemortem diagnostic testing to confirm MC intoxication cases is crucial for providing optimal supportive care and mitigating MC exposure.

Leticia Díez-Quijada et al. [9] explored the genotoxic effects of MC-LR and CYN combinations in rats. Their results revealed an increase in micronucleous formation in bone marrow. However, no DNA strand breaks nor oxidative DNA damage were induced, as shown in the comet assays. The histopathological study indicated alterations only in the highest dose group. Therefore, the combined exposure to cyanotoxins may induce genotoxic and histopathological damage in vivo.

Finally, two articles pointed out that MC-LR exacerbated the severity of illnesses such as non-alcoholic fatty liver disease [10] and dextran sulfate sodium (DSS)-induced colitis [11] in animal models. This is an important aspect, as the exposure to MC-LR, even at levels that are below the no observed adverse effect level (NOAEL) established in healthy animals, can worsen pre-existing pathologies.

All these studies have contributed to extend the knowledge on cyanotoxins and complete those published in our previous special issue, “Cyanobacteria and Cyanotoxins: New Advances and Future Challenges” [12]. Moreover, the 20 special issues dealing with this topic published thus far in the journal *Toxins* demonstrate the interest cyanobacteria and cyanotoxins have in the scientific community.
